# Exploring the Habitability of the Outer Solar System Icy Moons for the Extremotolerant Yeast *Rhodotorula frigidalcoholis*

**DOI:** 10.1111/1462-2920.70260

**Published:** 2026-02-24

**Authors:** Tommaso Zaccaria, Xuehui He, Kristina Beblo‐Vranesevic, Mihai G. Netea, Marien I. de Jonge, Petra Rettberg

**Affiliations:** ^1^ Applied Aerospace Biology Department, Institute of Aerospace Medicine German Aerospace Center (DLR) Cologne Germany; ^2^ Department of Internal Medicine Radboud University Medical Center Nijmegen the Netherlands; ^3^ Department of Laboratory Medicine, Laboratory of Medical Immunology Radboud University Medical Center Nijmegen the Netherlands; ^4^ Radboud Community for Infectious Diseases Radboud University Medical Center Nijmegen the Netherlands; ^5^ Department for Immunology and Metabolism, Life and Medical Sciences Institute (LIMES) University of Bonn Bonn Germany; ^6^ Radiation Biology Department, Institute of Aerospace Medicine German Aerospace Center (DLR) Cologne Germany

**Keywords:** icy moons, planetary protection, psychrotolerant, transcriptomics, yeast

## Abstract

Psychrophilic and psychrotolerant microorganisms have the unique ability to grow below 0°C, which makes them ideal candidates for studying how life could survive on the icy moons of the solar system. The renewed interest, in view of the JUICE and Europe Clipper missions, to explore these locations has pushed for the identification of organisms which could survive on the icy moons. In this study we selected the extremophilic yeast *Rhodotorula frigidalcoholis* given its innate ability to grow between 30°C and −10°C and to survive a range of extreme conditions including x‐ray, UV‐C and polychromatic UV radiation, desiccation at different temperatures, and freeze–thaw cycles. We report the survival of *R. frigidalcoholis* under conditions analogous to those found on icy moons. Using transcriptomic approaches, we present novel insights into differential gene expression before, during, and after exposure to combined icy moon conditions. We also identified the rapid activation of genes involved in catalytic activity and DNA repair during exposure. Our results contribute to a better understanding of the survival mechanisms of psychrotolerant microorganisms in extreme environments and can inform future life‐detection missions on moons such as Enceladus and Europa, also highlighting the need to consider yeasts in planetary protection efforts.

## Graphical abstract

We investigated the survival of the yeast *Rhodotorula frigidalcoholis* and analysed its RNA after exposure to icy moon conditions. Our results show how the yeast can tolerate the extreme conditions and performs metabolic functions under the exposure conditions; for example, by halting the cell cycle in order to repair. Created in BioRender. Zaccaria (2026): https://BioRender.com/h5uzc5g.

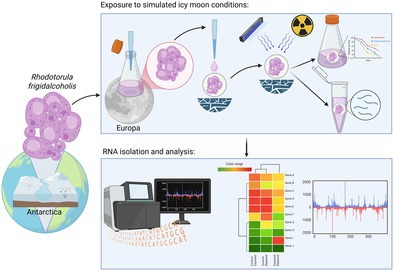

**Abbreviations**
COG: Cluster of Ortholog Groups
DEG: Differentially Expressed Genes
DGEA: Differential Gene Expression Analysis
ESA: European Space Agency
GO: Gene Ontology
GSEA: Gene Set Enrichment Analysis
ISS: International Space Station
JUICE: Jupiter Icy Moon Explorer
NASA: National Aeronautics and Space Administration
PCA: Principal Component Analysis
STAR: Spliced Transcripts Alignment to a Reference

## 1 Introduction

Exploring survival mechanisms for microbial life on the icy moons would improve our understanding of how these locations could support life. The renewed interest in solar system locations that may harbour life has been supported by ESA's JUICE and NASA's Europa Clipper missions currently on their way to the Jupiter system (Klenner et al. 2024). Investigations in the potential survival of microorganisms to the conditions of the icy moons can help elucidate risks associated with the unwanted contamination with biological material from Earth. Despite stringent decontamination strategies, forward contaminating risks remain, as elucidated by the COSPAR planetary protection community (Coustenis et al. 2023). Extensive astrobiology research has investigated the survival and adaptation of extremophilic microorganisms exposed to simulated planetary conditions. However, the number of studies focusing on yeasts are limited as compared to studies evaluating prokaryotic microorganisms (Leo and Onofri 2023). While yeasts have been included in a few spaceflight experiments, most of these have concentrated on *Saccharomyces cerevisiae* (Santa Maria et al. 2023). Nonetheless, important fungal studies in astrobiology have demonstrated that interest in other yeast and filamentous fungal species is increasing. Examples include the cultivation of *Cladosporium sphaerospermum* aboard the international space station (ISS), the survival of *Rhinocladiella similis* in Mars‐like perchlorate environments, or the exposure of the Antarctic *Cryomyces antarcticus* exposed to different ISS experiments (Averesch et al. 2022; Dos Santos, Schultz, Souza, et al. 2025; Schultz et al. 2023). The remarkable resilience of fungal microorganisms, compared to other eukaryotic organisms, is well documented in terrestrial extreme environments and under space conditions (Corbu et al. 2023; Deshevaya et al. 2024; Gostincar et al. 2010). Furthermore, the growing interest in using fungi for in situ resource utilisation (ISRU) on the Moon and Mars, further supports their relevance as promising model systems for astrobiology research (Cortesão et al. 2020; Figueira et al. 2025; Simões et al. 2023). Therefore, investigating other yeast species, particularly psychrotolerant species, which might be better adapted to surviving in extreme environments, is both timely and scientifically warranted.

Icy moons such as Saturn's Enceladus and Jupiter's Europa are celestial bodies with a frozen surface that overlay subsurface oceans, which may resemble those found on Earth (Kvorka and Čadek 2024). The Cassini‐Huygens mission played a crucial role in characterising these environments by analysing material ejected from the moon's surface by cold plumes (Waite et al. 2017). Additional factors contributing to the potential habitability of these moons include a kilometre thick ice shell that shields the subsurface ocean from the radiation‐rich external environment as well as internal heating of the ocean (Tjoa et al. 2020). The mechanism underlying this heating remains under debate, with two leading hypotheses: tidal heating generated by the moons' orbital interactions with their host planets, and the presence of hydrothermal vents (Bire et al. 2023; Tjoa et al. 2020), or possibly both.

Given the environmental conditions of icy moons, psychrophilic and psychrotolerant microorganisms are ideal candidates to investigate the potential for life and proliferate in such environments, due to their ability to grow at temperatures below the freezing point of water. Arctic and Antarctic locations, known to harbour diverse microbial communities, have recently been shown to contain high numbers of fungal species, highlighting the adaptability of such microorganisms to extreme conditions (De Menezes et al. 2020). Investigating the metabolism of these organisms under simulated icy moon conditions can provide critical insights into their survival strategies and inform organism‐specific handling protocols for space missions.

The yeast *Rhodotorula frigidalcoholis* (formerly JG‐1b) is a psychrotolerant organism isolated from Antarctic environments, capable of growing at temperatures below 0°C (Mykytczuk et al. 2013; Goordial et al. 2016). This yeast has demonstrated tolerance to a range of extreme conditions following cultivation in minimal media, including x‐ray radiation, UV‐C and polychromatic UV radiation, desiccation and freeze–thaw cycles (Zaccaria et al. 2026). Our research indicates that *R. frigidalcoholis* exhibits more tolerance to extreme conditions compared to several bacterial species. It has also been hypothesised that carotenoid pigment production, common among *Rhodotorula* species, may contribute to radiation resistance, particularly given the high UV flux in its native Antarctic habitat (Sharma and Ghoshal 2021). Furthermore, transcriptomic analysis by Touchette et al. (2022) revealed metabolic changes in *R. frigidalcoholis* when grown at 0°C versus 23°C. These findings position *R. frigidalcoholis* as a strong model organism for investigating metabolic responses under simulated icy moon conditions. Therefore, the aim of our study is to determine the survival limits of *R. frigidalcoholis* exposed to multifactorial stressors (desiccation, polychromatic UV, x‐ray) simulating icy moon conditions and to investigate its transcriptomic response to elucidate strategies for survival.

## 2 Experimental Procedures

### 2.1 Sample Growth

The yeast strain was kindly provided by Prof. Dr. Whyte from McGill University, Montreal, Canada. The organism was first grown in Universal Media for yeasts (UMY), containing 3.0 g yeast extract, 3.0 g malt extract, 5.0 g peptone, 10.0 g glucose in 1 L MQ water, at 25°C with orbital shaking at 125 rpm until late‐exponential phase. The organism was then inoculated in M9‐complete minimal salts medium containing: 47.8 mM Na_2_HPO_4_, 22.0 mM KH_2_PO_4_, 8.6 mM NaCl, 3.7 mM NH_4_Cl, 2 mM MgSO_4_, 0.1 mM CaCl_2_ and 0.02 mM Fe(III)Cl_3_. The medium was supplemented with 0.2% (w/v) of l‐glutamic acid (C_5_H_9_NO_4_) as carbon source, purchased from Sigma‐Aldrich; the media will be mentioned as M9‐glutamic from this point.

### 2.2 Sample Exposure

Three samples with four biological replicates were used for the exposure to icy moon conditions, and all exposure conditions were performed at room temperature, including desiccation, polychromatic UV and x‐ray radiation. This included control (growth in M9‐glutamic), exposed (growth in M9 and exposure to the icy moon simulated conditions) and repaired (all the previous with a 4‐h incubation step post‐exposure). Determination of the survival of the microorganism to single conditions was performed as per Zaccaria et al. (2026). After growth in M9‐glutamic, 200 μL of yeast culture was placed on sterile 1 cm glass disks. The disks were allowed to dry oxically at room temperature (relative humidity of 40% ± 10%) overnight under sterile conditions. The disks were stored for 7 days under the same and sterile conditions. UV exposure involved placing the disks under the 500S irradiation source (Dr. Hoenle AG; UV‐Technologie, Germany) for exposure to polychromatic UV radiation. Prior to sample irradiation, the UV source was allowed to warm up for > 30 min to allow the lamp to reach the full UV spectrum. UV fluence to reach the desired fluence was measured with the Bentham DMc150 transportable spectroradiometer (Bentham Instruments Ltd., Reading, United Kingdom), and sample exposure time was determined prior to the start of each experiment. The UV fluence during the exposure of the samples was measured at 20.838 W/m^2^ for a 100 cm distance from the light source for all the dose ranges of 500, 1000, 2500, 5000, and 7500 J/m^2^.

Following UV exposure, the disks were transferred to the Gulmay RS 225A (Gulmay Medical Ltd.) X‐ray source for exposure to ionising radiation. The output of the x‐ray source was set to 200 kV and 15 mA with the samples at a 10 cm distance below the source. The output of the source was measured with a UNIDOS dosimeter (PTW Freiburg, Germany) which registered 30 ± 5 Gy/min. After desiccation, UV and x‐ray exposure, the glass disks with the yeast samples were resuspended by vortexing in a 5 mL Eppendorf tube containing 1 mL of sterile PBS (4 g NaCl, 3 g KH_2_PO_4_ and 7 g Na_2_HPO_4_ in 1 L MQ water) for 30 s. The resuspended cells were used for dilution plating to determine survival via CFU counts and for the isolation of RNA.

**FIGURE 1:**
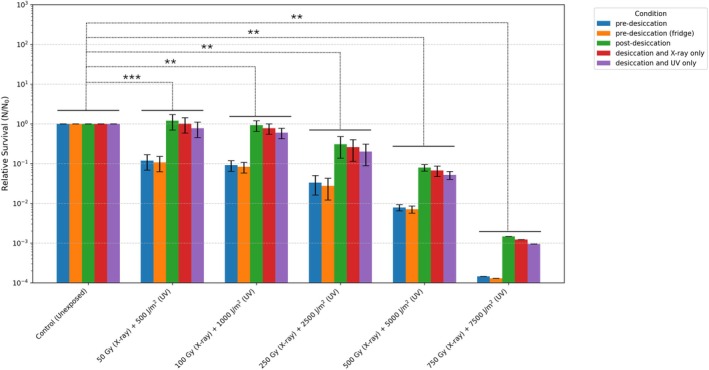
Survival of *R. frigidalcoholis* exposed to combined desiccation, x‐ray and polychromatic UV exposure conditions with different control conditions including without desiccation and storage at room temperature for the same period of time as desiccation (blue), with storage at 6°C for the same period of time as the desiccation condition (orange), with desiccation (green), excluding UV radiation (red), and excluding x‐ray radiation (purple). *n* = 3–6 biological replicates from three independent experiments, **p < 0.01, ***p < 0.001 *t*‐test: Two‐Sample Assuming Unequal Variances.

To analyse the RNA and determine the growth kinetics of the yeast post‐exposure, cells were resuspended in a sterile Erlenmeyer flask containing 100 mL of M9‐glutamic at 25°C. The growth kinetics of the yeast during incubation was determined by serial dilution plating and CFU counts every hour after incubation. The identification of increased CFU counts, and thus cell duplication, after 5 h, Figure 1, informed the selection of 4 h as the appropriate timepoint for RNA isolation in the Repair condition. This was performed in order to critically select a timepoint prior to the onset of cell division to avoid capturing the transcripts of the new cell generation instead of those actively recovering from exposure.

### 2.3 RNA Isolation and Quality Control

RNA was isolated following the phenol‐chloroform protocol from Jeffrey and Stuecker (2017). Following RNA isolation, electropherogram measurements were performed in order to ensure RNA quality. The Agilent 4200 TapeStation (Agilent Technologies Deutschland GmbH) with the Agilent High Sensitivity RNA ScreenTape was used to measure RNA concentration, eRIN values and to visually identify the 18S and 28S peaks prior to RNA sequencing.

### 2.4 RNA Shipping

Following extraction, the RNA samples were shipped in dry ice overnight to GENEWIZ Germany GmbH, Leipzig, Germany for RNA sequencing and preliminary analysis.

### 2.5 RNA Sequencing for Pathway Assessment/Bioinformatics

Next generation Illumina sequencing with 30 million reads per sample was performed by GENEWIZ Germany GmbH, Leipzig.

### 2.6 Mapping Sequence Reads to the Reference Genome

Sequence reads were trimmed to remove possible adapter sequences and nucleotides with poor quality using Trimmomatic v.0.36. The trimmed reads were mapped to the Rhodotorula_JG‐1b (GCA_001541205.1_Rhosp1) reference genome available on EnsemblFungi (https://ftp.ensemblgenomes.ebi.ac.uk/pub/fungi/release‐61/fasta/fungi_basidiomycota1_collection/rhodotorula_sp_jg_1b_gca_001541205/cds/) using the STAR aligner v.2.5.2b (https://www.ncbi.nlm.nih.gov/Traces/wgs/LQXB01).

### 2.7 Extracting Gene Hit Counts

Unique gene hit counts were calculated by using featureCounts from the Subread package v.1.5.2. Only unique reads that fell within exon regions were counted.

### 2.8 Differential Gene Expression Analysis

After extraction of gene hit counts, downstream differential gene expression analysis (DGEA) was performed using DESeq2, which is a comparison of gene expression between the selected groups of samples, summarised in Table 1. The Wald test was used to generate *p*‐values and log_2_ fold changes. Genes with an adjusted *p*‐value < 0.05 and absolute log_2_ fold change > 1 were called as differentially expressed genes (DEGs) for each comparison. Further filtering for known genes was performed in order to exclude unknown or hypothetical proteins.

**TABLE 1:** Sample grouping for differential gene expression analysis.

Sample label (replicate)	Sample description	Grouping for DGEA
A	Controls	Control (Group E)
B		
C		
D		
IA	Exposed to 7 days of desiccation, 750 Gy and 7500 J/m^2^	Exposed (Group F)
IB		
IC		
ID		
RA	Exposed and allowed to repair for 4 h at 25°C in liquid media	Repaired (Group G)
RB		
RC		
RD		

To visualise global transcriptional patterns, volcano plots were created to visualise the significant (adjusted *p*‐value < 0.05) upregulated and downregulated genes for each sample. This was performed by plotting on the *y*‐axis of log_10_ adjusted *p*‐value and the log_2_ fold change on the *x*‐axis. Furthermore, to visualise differences between samples, principal component analysis (PCA) plots were generated. First by standardising the data in order to normalise the gene expression levels, then by reducing the data to two principal components and plotting the scatterplot.

To understand the distribution of functional genes in clusters of ortholog groups (COG, Table 2), in the genome of *R. frigidalcoholis*, the genes of the reference genome were transformed into COG categories, using the NCBI database (https://ftp.ncbi.nlm.nih.gov/pub/COG/COG2024/data/) and plotted as a single bar chart. Furthermore, the DEGs identified by DGEA and grouped according to the samples described in Table 1 were also differentiated by COG categories and plotted in a bar chart to evaluate the distribution of functional genes by ortholog groups.

**TABLE 2:** Summary, label and description of COG (cluster of ortholog groups) categories extracted from Galperin et al. (2025).

Label	Description	Sub‐groups
J	Translational, ribosomal structure and biogenesis	Information storage and processing
A	RNA processing and modification	
K	Transcription	
L	Replication, recombination and repair	
B	Chromatin Structure and dynamics	
D	Cell cycle control, cell division, chromosome partitioning	Cellular processes and signalling
Y	Nuclear structure	
V	Defence mechanisms	
T	Signal transduction mechanisms	
M	Cell wall/membrane/envelope biogenesis	
N	Cell motility	
Z	Cytoskeleton	
W	Extracellular structures	
U	Intracellular trafficking, secretion, and vesicular transport	
O	Posttranslational modification, protein turnover, chaperones	
C	Energy production and conversion	Metabolism
G	Carbohydrate transport and metabolism	
E	Amino acid transport and metabolism	
F	Nucleotide transport and metabolism	
H	Coenzyme transport and metabolism	
I	Lipid transport and metabolism	
P	Inorganic ion transport and metabolism	
Q	Secondary metabolites biosynthesis, transport and catabolism	
R	General function prediction only	Poorly characterised
S	Function unknown	

To understand the number of shared DEGs between samples, a Venn diagram was generated to reflect these values as well as the number of shared up and down‐regulated DEGs. Additionally, the generation of a bar plot representing the COG category distribution of the shared genes was performed to evaluate shared functional metabolism for each sample.

Significant DEGs were analysed further with the gene set enrichment analysis (GSEA) of FungiFun3 (Garcia Lopez et al. 2024). The FungiFun3 tool was used to evaluate genome‐wide expression profiles of terms associated with *R. frigidalcoholis* and the DEGs identified from RNA‐seq analysis. The filtered DEGs were tested against the entire set of known genes as a background. Pathways were considered significant with an adjusted *p*‐value threshold (Benjamini–Hochberg correction) < 0.05 and were reported by plotting an enrichment score bar plot reporting which pathways have genes over or underrepresented. The reported values are normalised based on the differences in the sizes of the gene set and of the statistical background.

DEGs identified from all three sample comparisons were used to generate a clustered heatmap. To generate the hierarchically clustered heatmaps, the input data (gene expression log_2_ fold changes) were first normalised by *z*‐score using the following formula:

$$Z=\frac{X-\mu }{\sigma }$$

where *X* is the original value, *μ* is the mean of the column and *σ* is the standard deviation. Then the gene expression was compared between the three samples (Control vs. Exposed, Control vs. Repaired and Exposed vs. Repaired). Hierarchical clustering was performed separately on genes and on samples using Euclidian distance and average linkage computed with scipy.cluster.hierarchy of Seaborn 0.13.2. Dendrograms were set to represent cluster relationships. The Log_2_ fold change colour scale of the heatmap was set between +8 and −8.

Identification of resistance genes was performed by using the search function on UniProtKB and filtering the results by genes identified in the *R. frigidalcoholis* genome (UniProt 2025). If a gene was identified in the genome and was differentially expressed, it was reported in a table and as a clustered heatmap.

All the figures were generated using the Seaborn data visualisation library version 0.13.2 (Waskom 2021), which is an interface of Matplotlib (Hunter 2007). Unfiltered figures can be found in the Supporting Information.

## 3 Results

### 3.1 Survival to Combined Conditions

Previous research has shown the remarkable tolerance of *R. frigidalcoholis* to single extreme conditions (Zaccaria et al. 2026). Building on this, the yeast was subsequently exposed to a combination of stressors. Remarkably, it also survived combined exposure to desiccation, x‐ray and polychromatic UV (200–400 nm) radiation. The exposure regimens were applied in sequence to ensure that damage accumulated across treatments. All samples were exposed to 7 days of desiccation and increasing x‐ray and UV radiation regimens. The limit of survival was measured with 7 days of desiccation, 750 Gy of x‐ray radiation and 7500 J/m^2^ of polychromatic UV radiation (Figure 1).

**FIGURE 2:**
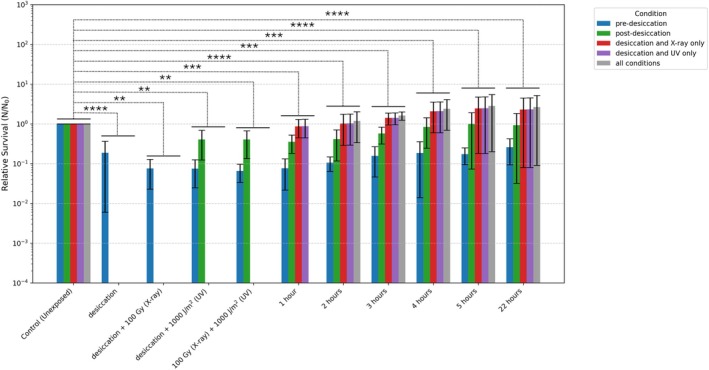
Growth of *R. frigidalcoholis* after exposure to the combined desiccation, x‐ray and polychromatic UV exposure conditions with different control conditions including without desiccation (blue), with desiccation (green), excluding UV radiation (red), excluding x‐ray radiation (purple) and with all the conditions combined (grey). *n* = 3–9 biological replicates from three independent experiments, **p < 0.01, ***p < 0.001, ****p < 0.0001 *t*‐test: Two‐Sample Assuming Unequal Variances.

To evaluate the metabolic changes required for survival, it was essential to ensure that *R. frigidalcoholis* could resume normal growth following exposure to combined conditions. Therefore, after exposure to the highest doses of combined conditions (7 days of desiccation, 750 Gy of x‐ray and 7500 J/m^2^ of polychromatic UV radiation), the desiccated samples were re‐suspended in PBS and incubated in minimal media at 25°C. An aliquot of the growing sample was taken every hour for 5 h and at 22 h to measure cell division by CFU plating (Figure 2). Based on the observed growth kinetics, a 4‐h post‐rehydration timepoint was chosen for RNA extraction.

### 3.2 Gene Expression Under Extreme Conditions

**FIGURE 3:**
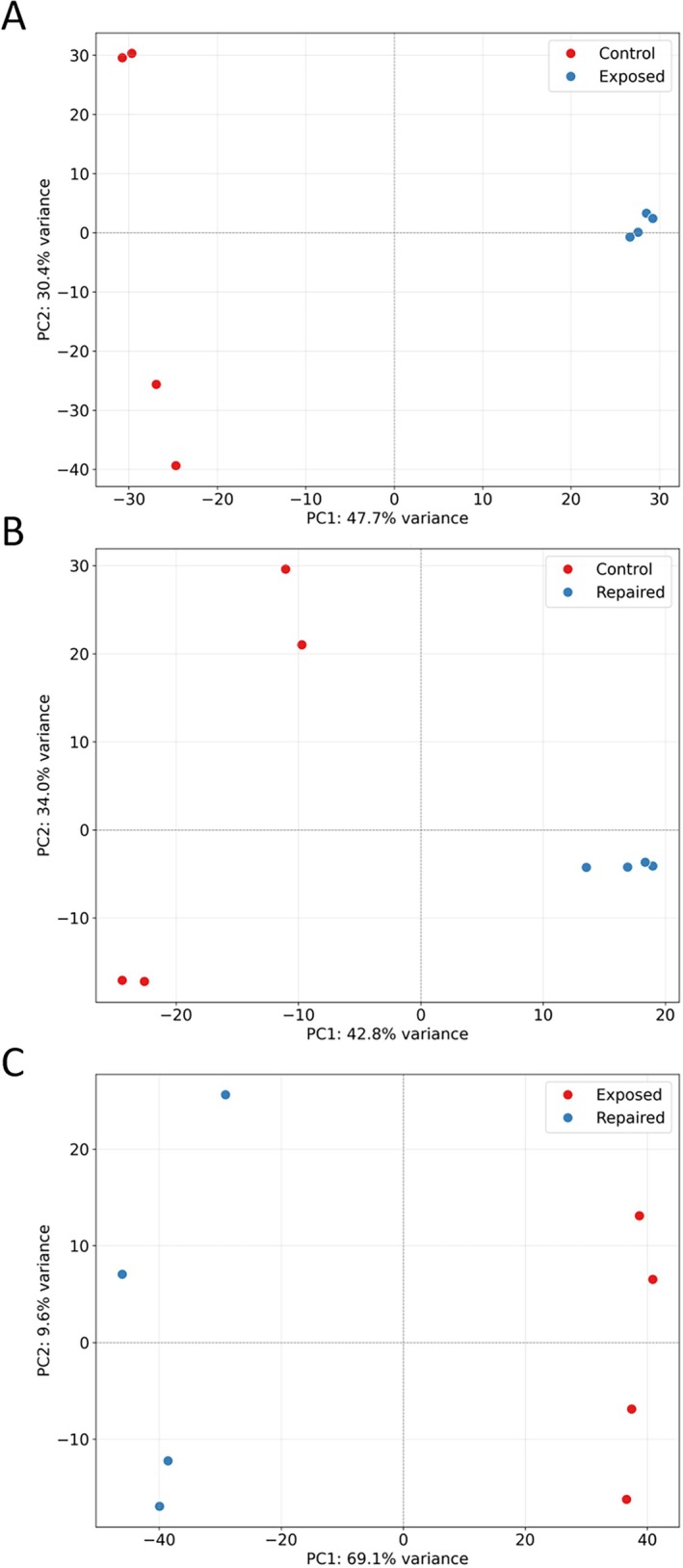
PCA plots showing the reproducibility within conditions and differences between them: Control vs. Exposed (A), Control vs. Repaired (B), and Exposed vs. Repaired (C).

To determine the reproducibility within conditions and differences between them, multiple principal component analyses (PCA) were performed. This revealed that the largest differences were found within the Exposed vs. Repaired comparison, with the strongest variance in PC1 (69%) between the two conditions (Figure 3C). However, the highest variances in PC2 were observed among the Control replicates (30%) (Figure 3A), and 34% (Figure 3B).

The total distribution of significant known DEGs (differentially expressed genes) for each comparison, as summarised in Table 3, showed a higher number of DEGs in the Control vs. Exposed and Exposed vs. Repaired comparisons than in the Control vs. Repaired comparison. Furthermore, except for the Exposed vs. Repaired, the number of downregulated DEGs exceeded the number of upregulated ones in the other two comparisons.

**TABLE 3:** Total number of upregulated and downregulated DEGs per comparison.

Comparison	Total DEGs	Upregulated DEGs	Downregulated DEGs
Control vs. Exposed	1653	799	854
Control vs. Repaired	726	273	453
Exposed vs. Repaired	2164	1119	1045

**FIGURE 4:**
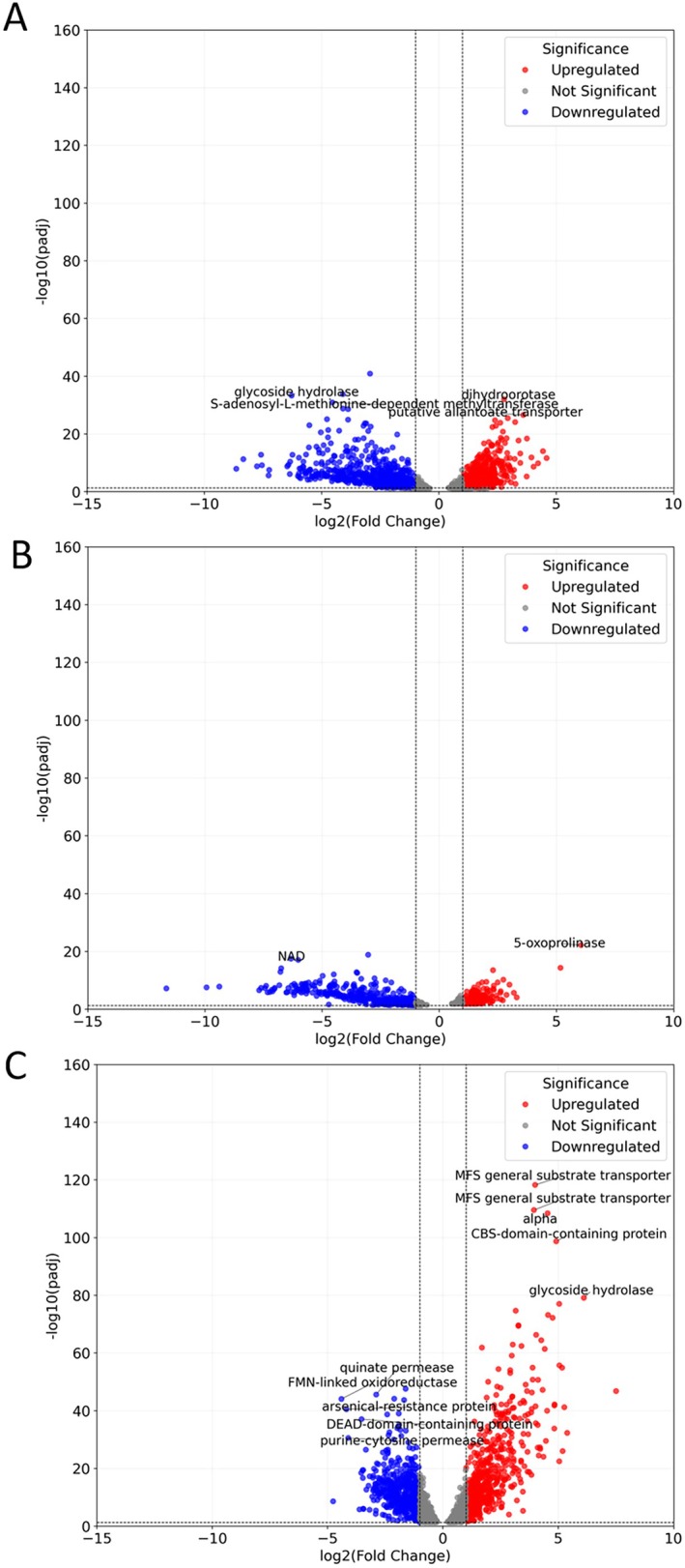
Volcano plots showing the significant distribution of upregulated (red dots) and downregulated (blue dots) DEGs for the samples Control vs. Exposed (A), Control vs. Repaired (B) and Exposed vs. Repaired (C).

Although the Exposed vs. Repaired comparison displays the highest total number of significant DEGs, the volcano plots show a narrower distribution of log_2_ fold changes compared to the Control vs. Exposed and Control vs. Repaired comparisons (Figure 4), indicating a lower diversity in gene expression changes but with a higher statistical significance. Nonetheless, Exposed vs. Repaired exhibits the broadest distribution of upregulated and downregulated DEGs, as based on −log_10_ (adjusted *p*‐value). When applying a ranking metric, it becomes evident that the top five most upregulated and downregulated genes are not consistently shared across all comparisons. However, a gene coding for a Major Facilitator Superfamily (MFS) substrate transporter, responsible for transporting a range of molecules across the cell membrane, consistently ranks among the most highly upregulated DEGs across all comparisons.

**FIGURE 5:**
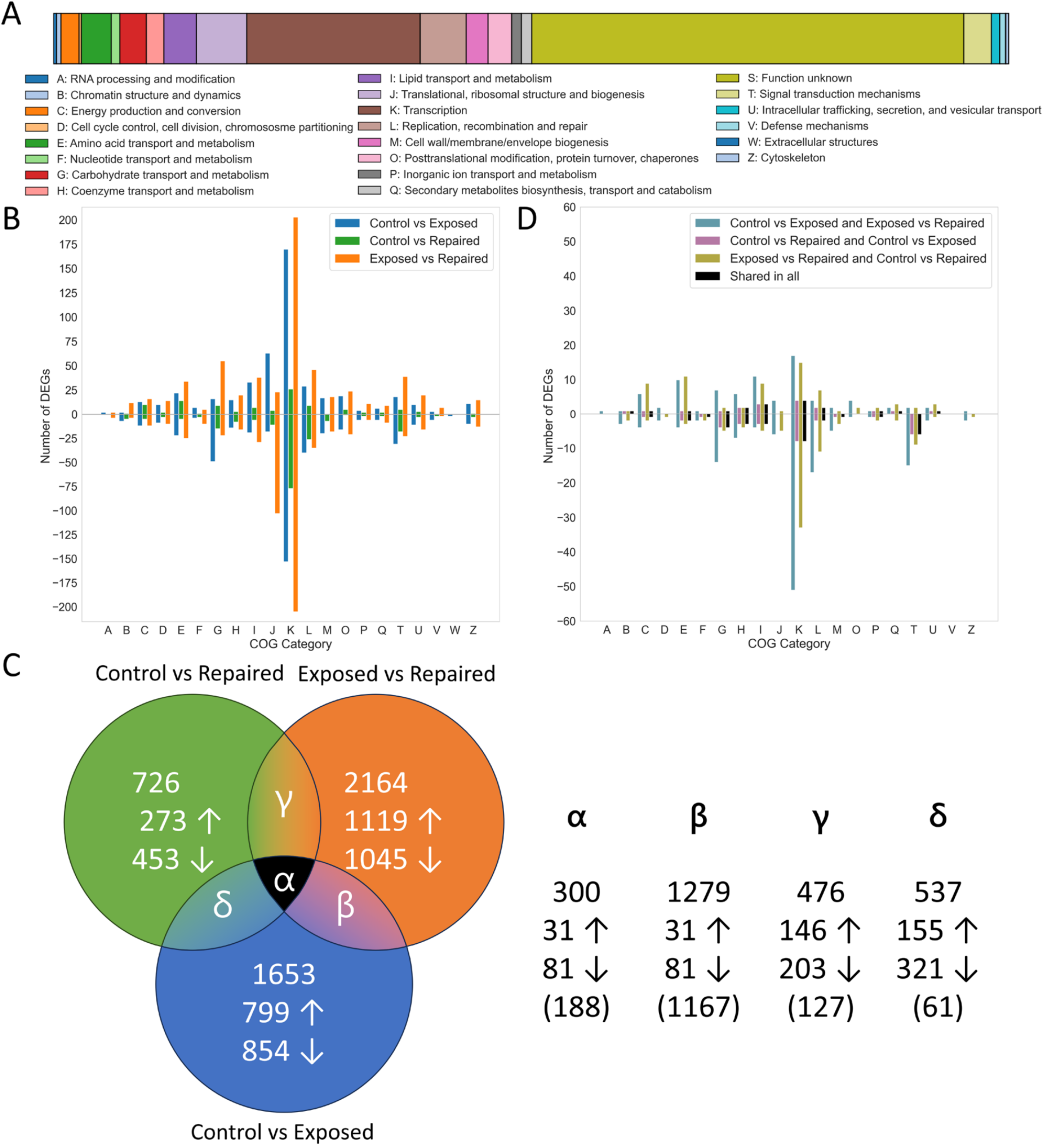
Distribution of COGs in the genome of *R. frigidalcoholis* (A). Frequency of upregulated and downregulated DEGs for each COG category under the different conditions. The total number of DEGs in each COG category that showed significant (*p* < 0.05) upregulation (> 1 log_2_ fold change) for Control vs. Exposed (Blue), Control vs. Repaired (Orange) and Exposed vs. Repaired (Green) (B). Distribution of unique and shared DEGs. Direction of arrows inside Venn diagram represent upregulation or downregulation. Numbers in brackets represent the DEGs which are shared between samples but are not upregulated or downregulated (C). COG category distribution for DEGs shared between samples. These include Control vs. Exposed and Exposed vs. Repaired (pink), Control vs. Repaired and Control vs. Exposed (cyan), Exposed vs. Repaired and Control vs. Repaired (yellow) and shared with all samples (black) (D).

The distribution of Cluster of Orthologous Groups (COGs) in the genome of *R. frigidalcoholis* revealed that a large proportion of genes have an unknown function (Figure 5A). Among the annotated genes, the majority are classified under category K, involved in transcription, followed by J, involved in translation, including ribosomal biogenesis. As expected, a high number of genes are also associated with lipid transport and metabolism (category I) and amino acid transport and metabolism (category E).

When examining the distribution of DEGs across the COG categories for the three pairwise comparisons, the highest number of both upregulated and downregulated genes is associated with transcription (Figure 5B). The relatively low number of DEGs observed in the Control vs. Repaired comparison (Table 4) is reflected by the shorter orange bars in the graph.

**TABLE 4:** Desiccation and radiation resistance ortholog genes found to be differentially expressed under the experimental conditions.

Resistance	Mechanism	Gene name	Function	Control vs. Exposed	Control vs. Repaired	Exposed vs. Repaired	References
Desiccation	Fatty acid oxidation	LPX1	Alpha/beta‐hydrolase	−5.59	−7.68	−1.95	Robison et al. (2024)
		LPX2	Alpha/beta‐hydrolase	−3.55	−3.36	2.01	
		POT1	Protection of telomeres protein 1 ssDNA‐binding domain‐containing protein	−3.49	−1.77	3.35	
	Osmolarity	hog1	Mitogen‐activated protein kinase hog1	0.55	5.67E‐6	1.37E‐5	Calahan et al. (2011)
		pbs2	MAP kinase	0.79	4.18E‐8	1.3E‐7	
		ssk2	Kinase‐like protein	−5.25	−3.36	2.01	
		ssk2	Kinase‐like protein	−4.99	−1.77	3.35	
		ssk2	STE/STE11 protein kinase	1.59	3.21E‐22	3.98E‐21	
		ssk2	Pkinase‐domain‐containing protein	−1.09	1.12E‐4	3.85E‐4	
		opy2	Membrane anchor Opy2 N‐terminal domain‐containing protein	1.26	1.03E‐5	4.58E‐5	
	Salinity	nst1	Stress response protein NST1	1.02	0.01	0.02	
	Trehalose	tps1	Trehalose‐6‐phosphate synthase component TPS1	−2.62	1.19E‐3	3.21E‐3	
		tps2	Family 20 glycosyltransferase	−1.93	0.01	0.02	
	Mediators and repressor	srb8	Mediator of RNA polymerase II transcription subunit 13	−2.48	2.8E‐6	1.41E‐5	
		pex10	RING‐type E3 ubiquitin transferase	1.31	2.25E‐5	9.16E‐5	
		pex10	RING‐type E3 ubiquitin transferase (cysteine targeting)	1.65	2.25E‐8	7.97E‐8	
		ras2	Small G‐protein Ras2	−1.43	8.92E‐4	2.5E‐3	
	Mediator of unfolded protein response	ire1	3‐phosphoinositide‐dependent protein kinase activity	0.50	4.77E‐3	0.01	Ren et al. (2020)
	Trehalose dispensability	nth1	DNA glycosylase	1.89	1.78E‐4	5.9E‐4	Tapia et al. (2015)
Radiation	Replication recombination repair	rad1	Rad1‐domain‐containing protein	−1.73	3.96E‐6	1.94E‐5	Bennett et al. (2001)
		rad10	DNA repair protein rad10	−0.78	0.03	0.06	
		rad18	RING‐type domain‐containing protein	−1.19	8.13E‐12	3.8E‐11	
		rad50	Rad50/SbcC‐type AAA domain‐containing protein	−1.15	0.01	0.02	
		rad50	RINT‐1 family protein	0.44	3.32E‐3	0.01	
		rad9	Rad9‐domain‐containing protein	−1.43	1.59E‐8	1.43E‐7	
		rad9	Histone‐lysine N‐methyltransferase, H3 lysine‐79 specific	−0.73	1.81E‐3	3.19E‐3	
		mec3	Checkpoint protein	0.80	3.5E‐3	0.01	
		mec3	Hus1‐like protein	0.73	0.01	0.01	
		rad17	p‐loop containing nucleoside triphosphate hydrolase protein	−1.13	4.74E‐4	1.42E‐3	
		rad17	Rad1‐domain‐containing protein	−1.73	3.96E‐6	1.94E‐5	
		ctf8	Transcription factor TFIIIC triple barrel domain‐containing protein	−0.88	0.01	0.01	
		chl1	ATP‐dependent DNA helicase CHL1	−1.65	1.21E‐3	3.27E‐3	
		chl1	DNA 5′‐3′ helicase	−1.45	1.15E‐3	3.14E‐3	
		ctf4		−1.79	0.01	0.01	
		dhh1	RNA helicase	1.28	1.95E‐3	4.98E‐3	
		nup133	Methyltransferase type 11	0.75	1.51E‐8	4.92E‐8	
		nup133	Nucleoporin‐domain‐containing protein	0.50	1.11E‐5	2.61E‐5	
		HPR1	THO complex subunit 1 transcription elongation factor‐domain‐containing protein	0.80	2.70E‐11	1.18E‐10	
		bem1	Bud emergence protein 1	1.19	9.25E‐4	2.58E‐3	
		nup84	SEC7 domain‐containing protein	0.67	1.07E‐8	3.56E‐8	
		rad17	P‐loop containing nucleoside triphosphate hydrolase protein	−1.13	4.74E‐4	1.42E‐3	
		rad17	Rad1‐domain‐containing protein	−1.73	3.96E‐6	1.94E‐5	
	Checkpoint	mec3	Hus1‐like protein	0.73	0.01	0.01	
	Nuclear pore complex	nup133	Methyltransferase type 11	0.75	1.51E‐8	4.92E‐8	
		nup133	Nucleoporin‐domain‐containing protein	0.50	1.11E‐5	2.61E‐5	
	Chromatin silencing telomers	ard1	Acyl‐CoA N‐acyltransferase	1.13	0.02	0.04	
		dhh1	DEAD‐domain‐containing protein	1.28	1.95E‐3	4.98E‐3	
	Mitotic chromosome transmission	chl1	DNA repair helicase	−1.65	1.21E‐3	3.27E‐3	
		chl1	DNA repair helicase	−1.45	1.15E‐3	3.14E‐3	
		pat1	Sm‐like ribonucleo protein	0.90	0.01	0.02	
	Ubiquitin degradation pathway	ubr1	Metallo‐hydrolase	0.82	2.31E‐4	4.63E‐4	
		doc1	Anaphase‐promoting complex	−1.24	0.01	0.02	
	Transcription/RNA metabolism	rpb9	DNA‐directed RNA polymerase II subunit I	1.82	2.99E‐4	9.38E‐4	
		ccr4	Putative Rpc11‐DNA‐directed RNA polymerase III subunit C11	1.41	0.01	0.03	
		ccr4	Putative RPA12‐13	2.27	8.05E‐9	7.91E‐8	
		ccr4	Not1‐domain‐containing protein	0.85	9.57E‐6	2.25E‐5	
		ccr4	CAF1‐domain‐containing protein	1.13	1.85E‐6	9.61E‐6	
		ccr4	Rcd1‐like protein	1.98	1.00E‐7	7.47E‐7	
		ccr4	Endonuclease	−0.75	0.02	0.04	
	Cytokinesis cytoskeleton spindle	rvs161	BAR‐domain‐containing protein	1.00	9.27E‐8	2.77E‐7	
		sac6	Putative SAC6‐Actin filament bundling protein	0.95	2.40E‐6	6.11E‐6	
	Cell wall heat shock	pdr13	HSP70‐domain‐containing protein	2.27	6.89E‐4	1.98E‐3	
		bck1	Pkinase‐domain‐containing protein	−1.09	1.12E‐4	3.85E‐4	
	Karyogamy	zuo1	DnaJ‐domain‐containing protein	−2.36	0.01	0.02	
		zuo1	DnaJ‐domain‐containing protein	2.30	3.19E‐3	0.01	
	Sterol metabolism	erg28	Erg28‐like protein	0.89	1.37E‐3	2.47E‐3	
	Radioresistance	mre11	Rad50/SbcC‐type AAA domain‐containing protein	−1.15	0.01	0.02	Wu et al. (2023)
		mre11	BRCT domain‐containing protein	−1.12	0.03	0.05	
	Pigment	N/A	Ferritin‐like domain‐containing protein	−1.78	0.01	0.02	
		N/A	p‐loop containing nucleoside triphosphate hydrolase protein	−3.49	5.89E‐5	2.19E‐4	
	SOS response	N/A	DUF159‐domain‐containing protein	−1.18	2.36E‐3	0.01	
	DNA damage sensor	Ku70/80	ATP‐dependent DNA helicase II subunit 1	0.71	0.05	0.08	
		Ku70/80	ATP‐dependent DNA helicase II subunit 2	−1.03	0.05	0.08	

The distribution trend of up‐ and downregulated DEGs across COG categories is uneven for each comparison, with very few categories showing a balanced number of genes in both directions. For example, in the Control vs. Exposed comparison, more upregulated genes fall within category J, that is, translation, ribosomal structure and biogenesis, while in the Exposed vs. Repaired comparison, this trend is reversed, with more genes being downregulated in the same category.

Interestingly, certain COGs, such as coenzyme transport and metabolism (H), cell wall/membrane biogenesis (M), posttranslational modification, protein turnover, chaperones (O), and intracellular trafficking, secretion, and vesicular transport (U), show a nearly equal distribution of up‐ and downregulated genes in both Control vs. Exposed and Exposed vs. Repaired comparisons. This suggests that the expression of genes in these categories may be consistently regulated during exposure and recovery, highlighting key cellular processes actively engaged by the yeast during stress response.

To identify genes shared across conditions and to identify expression trends, pairwise comparisons of DEGs were performed (Figure 5C). This analysis showed that 300 DEGs are shared across all three comparisons. Among these, 31 genes are consistently upregulated and 81 genes are consistently downregulated, while the remaining 188 genes are shared but show opposing expression patterns. As expected, the comparison between Exposed vs. Repaired and Control vs. Exposed shows the highest number of shared genes, reflecting the higher total number of DEGs in these comparisons. Overall, among the shared genes, the majority are downregulated, consistent with the general expression trend observed across individual comparisons, exception for Exposed vs. Repaired, where the pattern is reversed.

Although the distribution of COG categories among the shared genes generally follows the overall trend observed across all DEGs, notable differences are apparent (Figure 5D). As with the full dataset, the highest number of shared genes is still associated with transcription (K). However, several COG categories are absent in certain comparisons. Specifically, exposure‐related genes not associated with RNA processing and modification (A), cell cycle control, cell division, chromosome partitioning (D), translation, ribosomal structure and biogenesis (J), post‐translational modification, protein turnover, chaperones (O), defence mechanisms (V) and cytoskeleton (Z). In contrast to the overall COG distribution across all DEGs, the shared genes exhibit a stronger downregulation trend, as shown in Figure 5D. This pattern indicates that many of the genes common across comparisons, even when not consistently regulated in the same direction, tend to be more frequently downregulated. Despite these differences in COG category frequencies, the functional profile of the shared genes remains largely similar to that of individual comparisons. Most are still associated with key metabolic and regulatory functions, particularly coenzyme transport and metabolism (H), signal transduction mechanisms (T) and carbohydrate transport and metabolism (G).

### 3.3 Gene Set Enrichment Analysis

**FIGURE 6:**
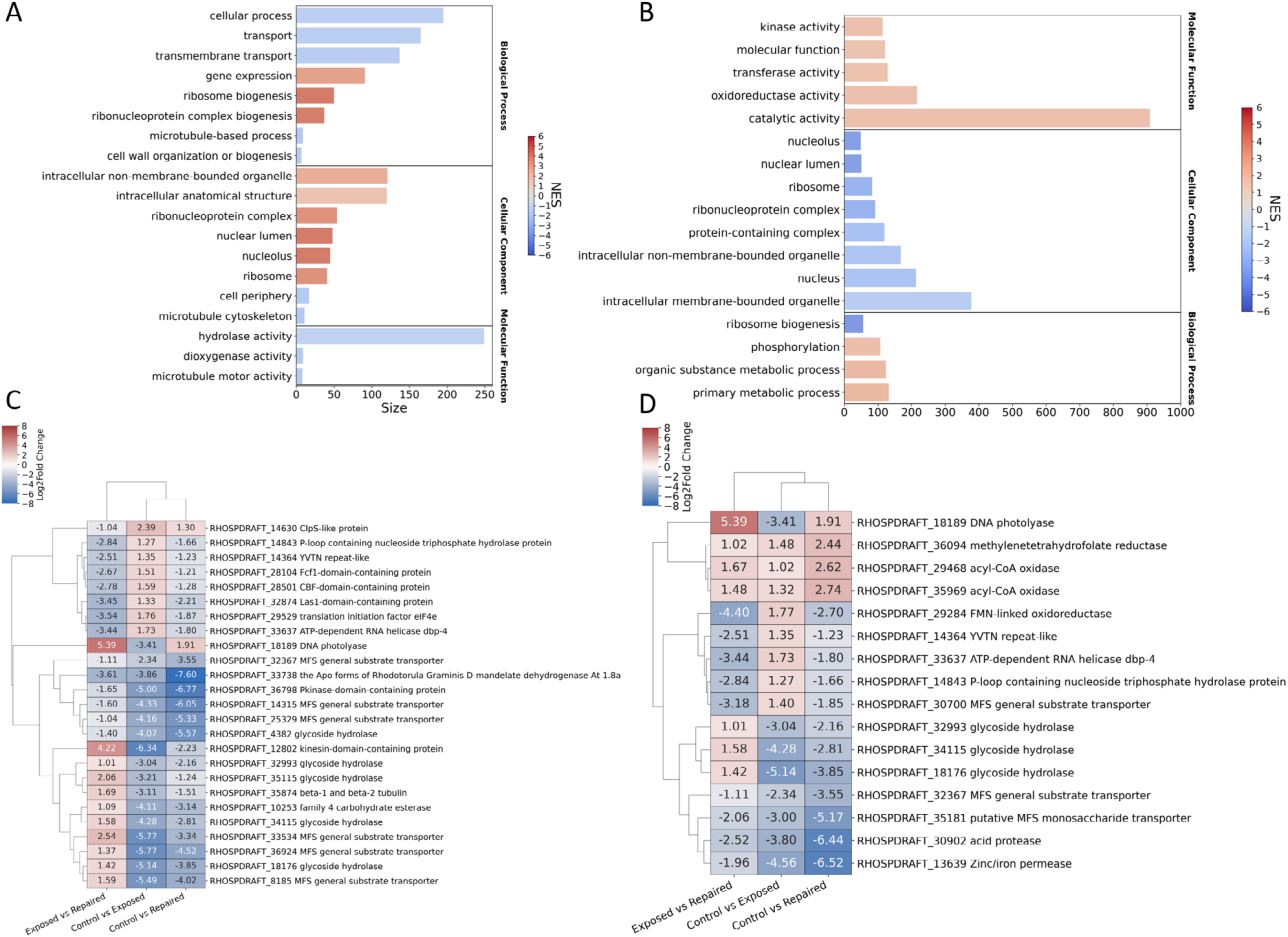
Gene Set Enrichment Analysis (GSEA) of the sample comparisons performed with FungiFun3. The figure summarises the enrichment of gene set pathways highlighting significant ones (*p*_adj_ < 0.05). The pathways are separated by ontology class (Biological process, Cellular component and Molecular function). Clustered heatmaps show the DEGs of interest for Control vs. Exposed (A and C) and Exposed vs. Repaired (B and D) comparisons.

Gene set Enrichment analysis (GSEA) using the FungiFun3 tool was performed to elucidate the over or underrepresented pathways between samples. The analysis revealed 94 pathways involved in the Control vs. Exposed comparison, 101 in Exposed vs. Repaired, Figure 6A,B and 35 pathways in Control vs. Repaired (Figure S2). The normalised enrichment score (NES) in Control vs. Exposed ranged between 4.11 and −2.02, and upregulated pathways reflected protein synthesis (ribosome biogenesis, nuclear lumen, nucleolus) while downregulated pathways are related to cell structure components (microtubule cytoskeleton, cell wall organisation) and biosynthetic processes (dioxygenase activity, microtubule motor activity) (Figure 6A). However, the strongly enriched up‐ and down‐regulated pathways had a lower gene count than ones with weaker enrichment (hydrolase activity, cellular process, intracellular non‐membrane‐bounded organelle, intracellular anatomical structure).

In contrast to Control vs. Exposed, the pathways identified in the Control vs. Repaired comparison were fewer, indicating similar functions being carried out by the yeast under the two conditions (Figure S2). This is also supported by the low number of genes employed in the pathways (anion binding) and the low enrichment score (amino acid synthesis). Nonetheless, Control vs. Repaired shows an overall positive enrichment, 30 out of 35 pathways being upregulated. The nature of the pathways in the comparison being associated to baseline activity can also be identified by their functions (amino acid biosynthetic process, oxidoreductase activity, anion binding, ligase activity).

The high number of pathways as well as the high number of pathways with a size > 100 genes in Exposed vs. Repaired reflects the difference in activity performed between the two conditions (Figure 6B). The GSEA also highlighted how catalytic activity is the pathway with the highest number of genes involved across all comparisons in this study. Despite a number of pathways identified in Control vs. Exposed, the reversal in NES range for Exposed vs. Repaired shows the difference in metabolic function between the two comparisons. Under this comparison, ribosome biogenesis and protein synthesis are downregulated (ribonucleoprotein complex, ribosome, ribosomal subunit, nucleolus) while catalysing and phosphorylating mechanisms, including DNA repair, are upregulated (organic substrate metabolic process, primary metabolic process, phosphorylation, transferase activity, kinase activity).

Generation of clustered heatmaps from the genes involved in the pathways highlighted by GSEA support our findings (Figure 6C,D), for further details see Figures S4–S6. For example, ribosome biogenesis and protein synthesis genes are upregulated in Control vs. Exposed only (Figure 6C) and the importance of DNA repair and hydrolyzing genes such as DNA photolyase and Alpha/beta hydrolase being shared with other species of the *Rhodotorula* genus. Gene clustering also revealed how a number of genes are shared across pathways (acyl‐CoA oxidase, glycoside hydrolase), underlining their role.

### 3.4 Desiccation and Radiation Resistance Gene Expression Patterns

Investigations into the *R. frigidalcoholis* genome using UniProtKB (UniProt 2025) revealed several gene orthologs which might support resistance to a number of extreme conditions. Such genes were identified in other organisms which showed tolerance to desiccation or radiation. The search yielded 72 of these “resistance genes” differentially expressed under all sample conditions, 53 of which are related to radiation and 19 to desiccation (Table 4). Interestingly, despite all the genes eliciting a response, only the Control vs. Exposed comparison showed higher log_2_ fold changes. Furthermore, under Control vs. Exposed, approximately half of the radiation and half of the desiccation resistance genes are upregulated. However, the DEGs with the highest log_2_ fold change can be seen at the top as RHOSPDRAFT_31273 (LPX1), RHOSPDRAFT_31279 Alpha/beta‐hydrolase (LPX2), and RHOSPDRAFT_33214 Protection of telomeres protein 1 ssDNA‐binding domain‐containing protein (POT1), clustered separately from all other DEGs and associated to desiccation tolerance. As a whole, these results support the GSEA analysis and further cement the understanding of how *R. frigidalcoholis* is metabolically active under the exposure conditions compared to Control or Repair.

## 4 Discussion

Earlier studies have shown the resilience of *R. frigidalcoholis*; particularly, they have highlighted its ability to grow at subzero temperatures and to tolerate extreme environmental conditions (Mykytczuk et al. 2013; Touchette et al. 2022). However, the survival and metabolic activity of this yeast and other microorganisms under icy moon‐like conditions remain largely unexplored (Spry et al. 2024). The multifactorial effects of various environmental stressor characteristics of icy moons on microbial survival are still poorly understood. This knowledge gap is particularly important given the upcoming end‐of‐mission plans for JUICE and Europa Clipper spacecraft, both of which are scheduled to crash on Ganymede, the largest moon of Jupiter and the entire solar system (Castelvecchi 2023). Despite Ganymede being chosen as a crash site for both ESA's and NASA's missions due to the assumption that its subsurface ocean does not interact with the surface, this end‐of‐mission decision remains subject to change if new evidence suggests otherwise. Therefore, it is crucial to investigate how icy moon conditions affect psychrotolerant microorganisms, which are the most likely terrestrial life forms to survive such environments. The selection of *R. frigidalcoholis* as a model organism is further supported by its intrinsic ability to adapt, by, for example, intracellular accumulation of osmolytes during high osmotic pressures, which occur during freezing of water, and to maintain membrane fluidity (Leo and Onofri 2023).

Fungal research in the context of space biology is growing; however, most of the investigations focus on the response of the microorganisms to radiation, largely because fungi are among the most radiation‐tolerant eukaryotes (Schultzhaus, Chen, et al. 2020). Most yeast research studies to date have centred on the well‐studied genus *Saccharomyces*, particularly *S. cerevisiae* . For example, *S. cerevisiae* has been reported to tolerate up to 60 Gy of particle galactic cosmic ray (GCR)‐like ionising radiation (Liddell et al. 2023). In contrast, our findings show that *R. frigidalcoholis* demonstrates significantly higher resistance to higher doses of ionising radiation, suggesting that it may be a more robust candidate for space survival under such conditions. Supporting the importance of testing multiple stressors, earlier research on *Saccharomyces cerevisiae* *var. ellipsoideus* reported survival after exposure to 750 Gy of γ‐radiation and brief heat shock at 60°C, highlighting the need to investigate synergistic stress effects (Petin and Kim 2004). Additionally, environmental yeast isolates such as *R. mucilaginosa* , *Dioszegia fristingensis* and *Cystofilobasidium macerans* have also been studied for their tolerance to UV radiation. However, these investigations did not specifically examine the impact of space‐relevant DNA‐damaging UV‐C radiation (Libkind et al. 2009). While desiccation tolerance has been investigated in some yeast species, those studies have typically shown limited survival and no growth under nutrient‐limited conditions (Ren et al. 2020). Nonetheless, other fungal candidates such as the black fungus *Cryomyces antarcticus* and *Rhinocladiella similis* have been used in studies to evaluate the extraterrestrial habitability of Mars through exposure to Fe particle radiation and interactions with Mars regolith simulants (Dos Santos, Schultz, Dal'rio, et al. 2025; Pacelli et al. 2021). Furthermore, proteomic analyses of the halotolerant yeast *Debaryomyces hansenii* have also shown its resilience to perchlorate stress under Martian brine conditions (Heinz et al. 2022). Taken together, our results demonstrate that *R. frigidalcoholis* is a highly promising (and potentially superior) candidate for space survival compared to other yeast species, due to its exceptional resistance to multiple space‐relevant stressors, including radiation, desiccation and nutrient limitation.

The genome of *S. cerevisiae* is circa 12 Mbp in size (Belda et al. 2019) and studies on the reference strain S288c have shown that only around 6% of its genes remain functionally uncharacterised (Dietrich et al. 2025). In contrast, although the genome of *R. frigidalcoholis* is larger in size, it contains 53% of genes with unknown function, underscoring how comparatively understudied this organism is (Goordial et al. 2016). This high number of unknown genes is reflected in our transcriptome analysis where a large fraction of DEGs lack known functional annotations. These unknown DEGs might play important roles in stress responses and survival under the simulated icy moon conditions, highlighting the need for future functional studies.

Among the known DEGs, the majority of both up‐ and downregulated genes are related to transcription and translation. This suggests that significant changes in these core processes occur during exposure and recovery. In particular, the Exposed vs. Repaired comparison showed strong downregulation of translation‐related genes, likely indicating temporary cell cycle arrest. Such an arrest is a known stress response. For instance, *Exophiala dermatitidis* halts cell division after γ‐irradiation to allow DNA repair before resuming growth (Schultzhaus, Romsdahl, et al. 2020). A similar mechanism may occur in *R. frigidalcoholis*, as evidenced by downregulation of genes involved in translation, ribosome biogenesis, and DNA replication during recovery, suggesting a coordinated response to preserve genomic integrity.

Concurrently, the upregulation of genes encoding for enzymes with catalytic activity in the Exposed vs. Repaired condition suggests that *R. frigidalcoholis* activates biochemical repair processes. The enzymes are essential for double‐strand break (DSB) repair in yeast and humans (Cullati et al. 2024). Increased expression of genes related to energy production further supports this, indicating that energy‐intensive repair processes are prioritised during stress. Similar responses have been observed in industrial yeasts during alcohol fermentation (Kitagaki and Takagi 2014).

Plasma membrane damage can also halt cell cycle progression. This includes damage from desiccation, ionising radiation, DNA damage such as pyrimidine dimers and DSBs, and the production of reactive oxygen species (ROS) triggered by both UV and ionising radiation (Aylon and Kupiec 2004; Clear et al. 2020; Wang et al. 2020; Gull et al. 2025). In *S. cerevisiae* , membrane damage has been directly linked to inhibition of DNA replication and activation of preservation mechanisms (García et al. 2004; Kono et al. 2016). Given the severity of the stressors applied in our experiments, likely *R. frigidalcoholis* experienced similar membrane disruption. Despite compositional differences, studies have shown that even lower x‐ray radiation doses than those used here can damage eukaryotic membranes (Wang et al. 2020).

Interestingly, despite the presumed need for signalling to initiate repair and arrest growth, we observed a significant downregulation of DEGs associated with signal transduction. This may indicate dysregulation of signalling pathways in *R. frigidalcoholis* under extreme stress. In *S. cerevisiae* , DNA damage checkpoints are known to delay mitosis allowing repair, which could explain our findings (Pardo et al. 2017; Zhou et al. 2024).

The experimental design aimed to capture activation of DNA repair pathways during recovery. Unexpectedly, several key repair genes were already upregulated during exposure, while the cells were desiccated. These include DNA photolyase, LPX2, POT1, RAD50, MRE11, Ku70, and Ku80. This suggests that the exposure induced both pyrimidine dimers and DSBs, supported by activation of both homologous recombination (RAD50, MRE11) and non‐homologous end joining (Ku70, Ku80) pathways (Bennett et al. 2001; Wu et al. 2023). The upregulation of DNA photolyase, which specifically repairs cyclobutene pyrimidine dimers, further supports this (Thiagarajan et al. 2011). Increased expression of *POT1*, a gene involved in telomere protection, underscores the cell's effort to maintain genomic stability (Shakirov et al. 2010). These mechanisms are critical, as faulty repair can lead to tumorigenesis in higher eukaryotes (Vassilev and Depamphilis 2017).

DNA photolyase is conserved across all domains of life (Essen and Klar 2006) and is activated by visible light between 300 and 500 nm (Thiagarajan et al. 2011). While Earth's hydrothermal vents primarily emit 750–1050 nm wavelengths, light in the 400–600 nm range has also been detected (Van Dover et al. 1996; White et al. 2002). Although insufficient for photolyase activation under Earth conditions, icy moon subsurface ocean chemistry might enable production of suitable light for in situ enzyme activity. Such environments, analogous to Earth's hydrothermal systems, could support life and have been proposed as potential sites for life's origin (Martin et al. 2008; Zierenberg et al. 2000). Thus, activation of repair mechanisms, including photolyase, in *R. frigidalcoholis* under extreme conditions supports its candidacy as a model organism for astrobiology and life detection missions. These findings also strengthen the case for the habitability of icy moons by psychrotolerant organisms.

Our study demonstrates that *R. frigidalcoholis* can tolerate icy moon‐like conditions and activates DNA repair and metabolic pathways even in a desiccated state. Like other microorganisms, it appears to prioritise repair to cell cycle progression. We also emphasise the need for improved genome annotation, as most DEGs have unknown functions. Limitations in our study include conducting experiments at room temperature, not evaluating the growth after freezing the yeast cells, a small number of RNA sampling timepoints, making it unclear whether certain responses occur during desiccation (within 24 h) or exposure (within 7 days). Additionally, comparisons with other yeasts are limited, as most reference data come from *S. cerevisiae* . We also recognise potential bias in selecting for desiccation‐ and radiation‐resistant genes.

Future studies should determine how rapidly *R. frigidalcoholis* activates DNA repair and whether this occurs under predicted in situ icy moon conditions. Data from upcoming missions like JUICE and Europa Clipper will be key in assessing true habitability. Multifactorial exposure studies, like ours, also offer valuable insights for planetary protection, informing strategies to prevent forward contamination. Ultimately, broader investigations across life domains are needed. Given the ethical and scientific imperative to protect extraterrestrial environments, particularly promising ones like icy moons, stringent contamination control is essential.

## Author Contributions

**Petra Rettberg and Marien I. de Jonge:** conceptualisation. **Petra Rettberg, Tommaso Zaccaria and Kristina Beblo‐Vranesevic:** methodology. **Tommaso Zaccaria and Xuheui He:** formal analysis and investigation. **Tommaso Zaccaria:** writing – original draft preparation. **Petra Rettberg, Kristina Beblo‐Vranesevic, Mihai G. Netea, Marien I. de Jonge and Xuehui He:** writing – review and editing. **Petra Rettberg and Mihai Netea:** funding acquisition. **Petra Rettberg and Kristina Beblo‐Vranesevic:** resources. **Petra Rettberg, Kristina Beblo‐Vranesevic, Mihai G. Netea and Marien I. de Jonge:** supervision.

## Funding

This work was supported by Deutsches Zentrum für Luft‐ und Raumfahrt.

## Conflicts of Interest

The authors declare no conflicts of interest.

## Supporting information


**Figure S1:** Shows the GSEA (Gene Set Enrichment Analysis) GO enrichment analysis performed with FungiFun3 for the Control vs. Exposed sample. The figure summarises the enrichment of each gene set and shows the significant ones *p*_adj_ < 0.05. The genes are separated by ontology class: Biological process, Cellular component and Molecular function. The colour of each bar represents the normalised enrichment score (NES) with for each pathway.
**Figure S2:** Shows the GSEA (Gene Set Enrichment Analysis) GO enrichment analysis performed with FungiFun3 for the Control vs. Repaired sample. The figure summarises the enrichment of each gene set and shows the significant ones *p*_adj_ < 0.05. The genes are separated by ontology class: Biological process, Cellular component and Molecular function. The colour of each bar represents the normalised enrichment score (NES) with for each pathway. *Oxidoreductase activity, acting on CH‐CH group of donors. +Oxidoreductase activity, acting on the CH—CH group of donors, oxygen as acceptor. #Oxidoreductase activity, acting on the CH—CH group of donors, with flavin as acceptor.
**Figure S3:** Shows the GSEA (Gene Set Enrichment Analysis) GO enrichment analysis performed with FungiFun3 for the Exposed vs. Repaired sample. The figure summarises the enrichment of each gene set and shows the significant ones *p*_adj_ < 0.05. The genes are separated by ontology class: Biological process, Cellular component and Molecular function. The colour of each bar represents the normalised enrichment score (NES) with for each pathway.
**Figure S4:** Clustered heatmap of the Control vs. Exposed of GO enrichment of the differentially regulated genes in the analysis by FungiFun3.
**Figure S5:** Clustered heatmap of the Control vs. Repaired of GO enrichment of the differentially regulated genes in the analysis by FungiFun3.
**Figure S6:** Clustered heatmap of the Exposed vs. Repaired of GO enrichment of the differentially regulated genes in the analysis by FungiFun3.

## Data Availability

The data that support the findings of this study are openly available in Mendeley Data at https://data.mendeley.com/datasets/m65mc3vd5w/1, reference number 10.17632/m65mc3vd5w.1.
