# Supplementary material for: Exploring the Habitability of the Outer Solar System Icy Moons for the Extremotolerant Yeast Rhodotorula frigidalcoholis
Source: Environ Microbiol. 2026 Feb 24;28(2):e70260. doi: 10.1111/1462-2920.70260 (PMC12930229; doi:10.1111/1462-2920.70260)
Supplement: Supplementary file 1 — Figure S1: Shows the GSEA (Gene Set Enrichment Analysis) GO enrichment analysis performed with FungiFun3 for the Control vs. Exposed sample. The figure summarises the enrichment of each gene set and shows the significant ones p adj < 0.05. The genes are separated by ontology class: Biological process, Cellular component and Molecular function. The colour of each bar represents the normalised enrichment score (NES) with for each pathway. Figure S2: Shows the GSEA (Gene Set Enrichment Analysis) GO enrichment analysis performed with FungiFun3 for the Control vs. Repaired sample. The figure summarises the enrichment of each gene set and shows the significant ones p adj < 0.05. The genes are separated by ontology class: Biological process, Cellular component and Molecular function. The colour of each bar represents the normalised enrichment score (NES) with for each pathway. *Oxidoreductase activity, acting on CH‐CH group of donors. +Oxidoreductase activity, acting on the CH—CH group of donors, oxygen as acceptor. #Oxidoreductase activity, acting on the CH—CH group of donors, with flavin as acceptor. Figure S3: Shows the GSEA (Gene Set Enrichment Analysis) GO enrichment analysis performed with FungiFun3 for the Exposed vs. Repaired sample. The figure summarises the enrichment of each gene set and shows the significant ones p adj < 0.05. The genes are separated by ontology class: Biological process, Cellular component and Molecular function. The colour of each bar represents the normalised enrichment score (NES) with for each pathway. Figure S4: Clustered heatmap of the Control vs. Exposed of GO enrichment of the differentially regulated genes in the analysis by FungiFun3. Figure S5: Clustered heatmap of the Control vs. Repaired of GO enrichment of the differentially regulated genes in the analysis by FungiFun3. Figure S6: Clustered heatmap of the Exposed vs. Repaired of GO enrichment of the differentially regulated genes in the analysis by FungiFun3. [file EMI-28-e70260-s001.docx]

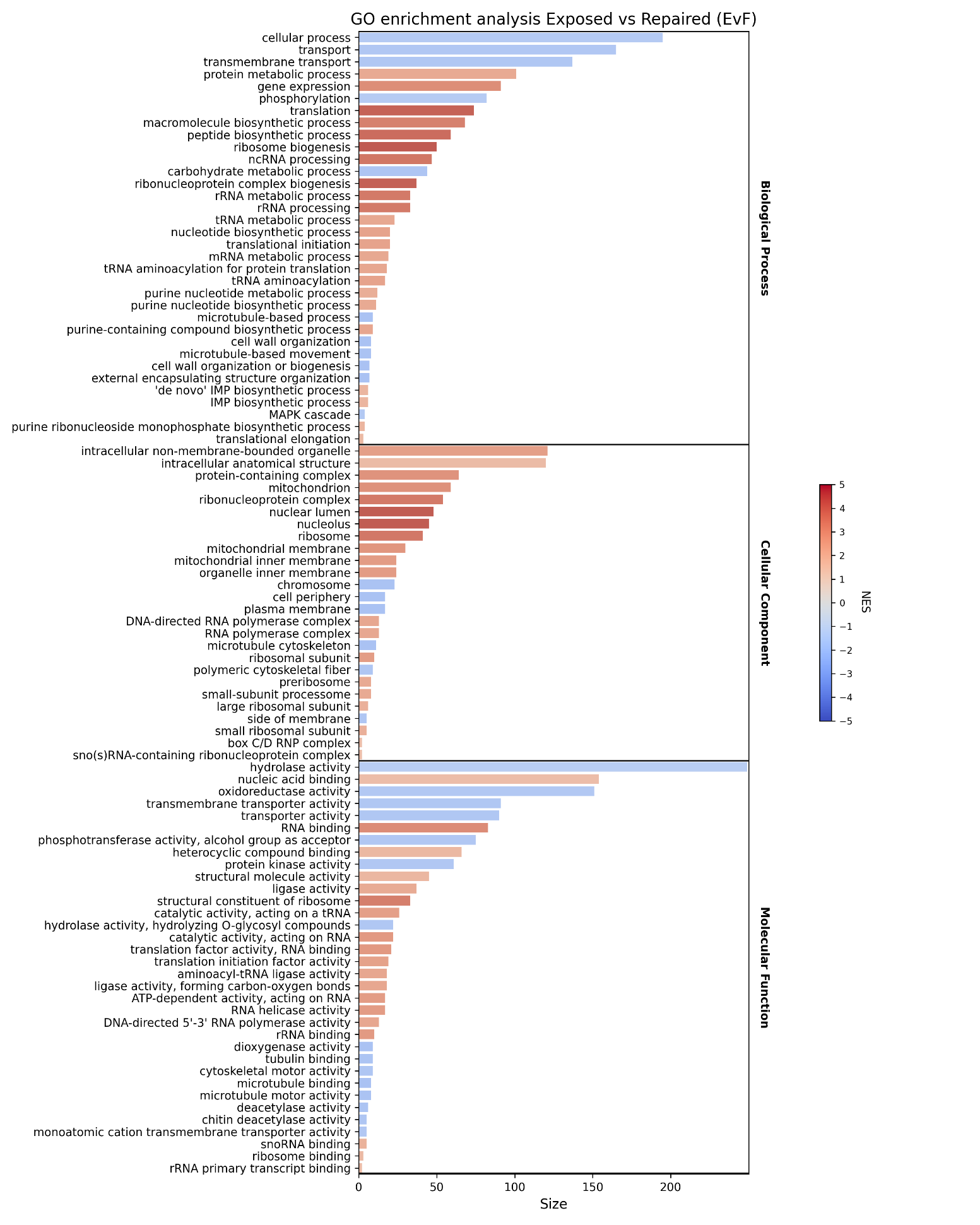


**Fig. S1** Shows the GSEA (Gene Set Enrichment Analysis) GO enrichment analysis performed with FungiFun3 for the Control vs Exposed sample. The figure summarizes the enrichment of each gene set and shows the significant ones padj<0.05. The genes are separated by ontology class: Biological process, Cellular component and Molecular function. The color of each bar represents the normalized enrichment score (NES) with for each pathway


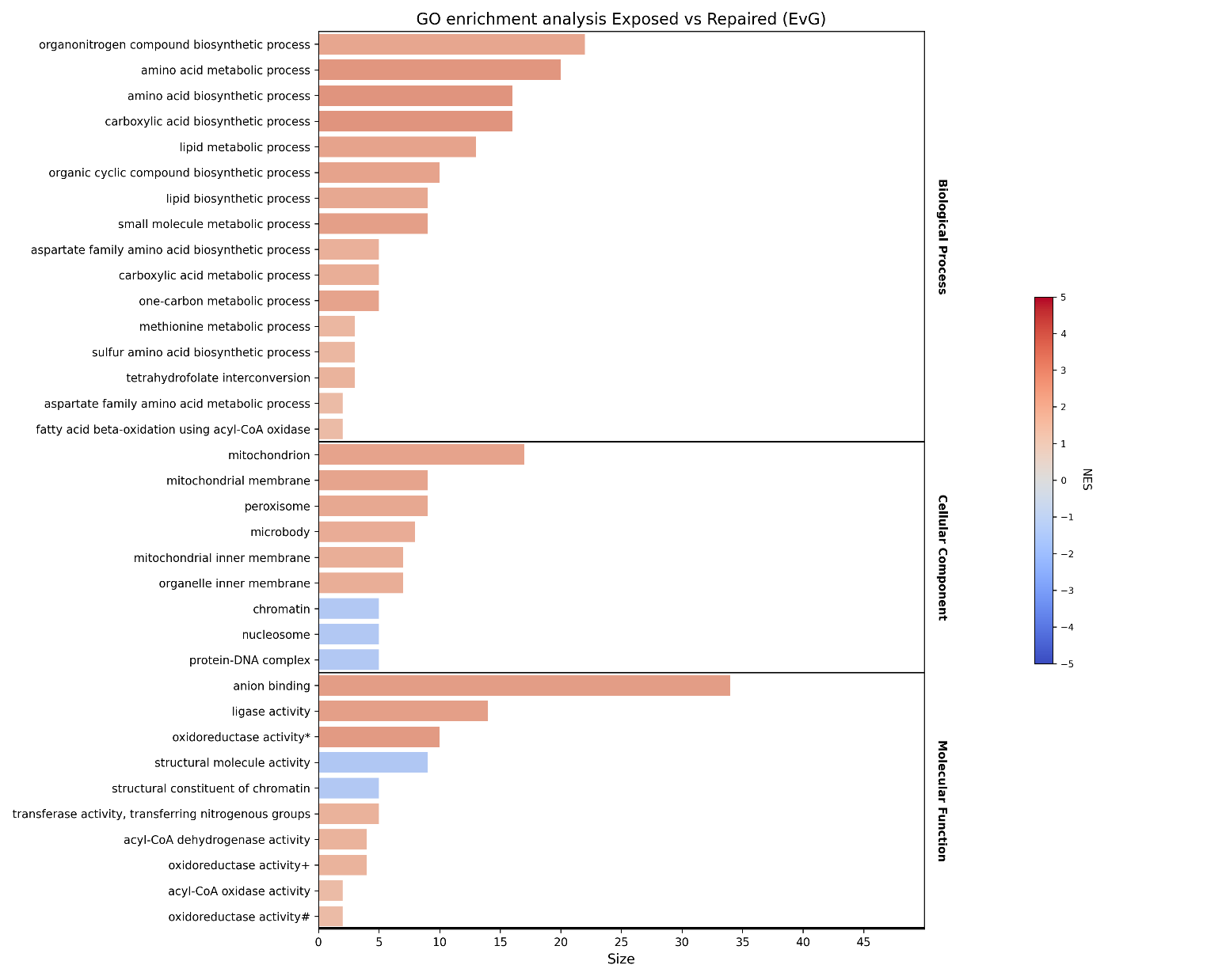


**Fig. S2** Shows the GSEA (Gene Set Enrichment Analysis) GO enrichment analysis performed with FungiFun3 for the Control vs Repaired sample. The figure summarizes the enrichment of each gene set and shows the significant ones padj<0.05. The genes are separated by ontology class: Biological process, Cellular component and Molecular function. The color of each bar represents the normalized enrichment score (NES) with for each pathway. *Oxidoreductase activity, acting on CH-CH group of donors. +Oxidoreductase activity, acting on the CH-CH group of donors, oxygen as acceptor. #Oxidoreductase activity, acting on the CH-CH group of donors, with flavin as acceptor.


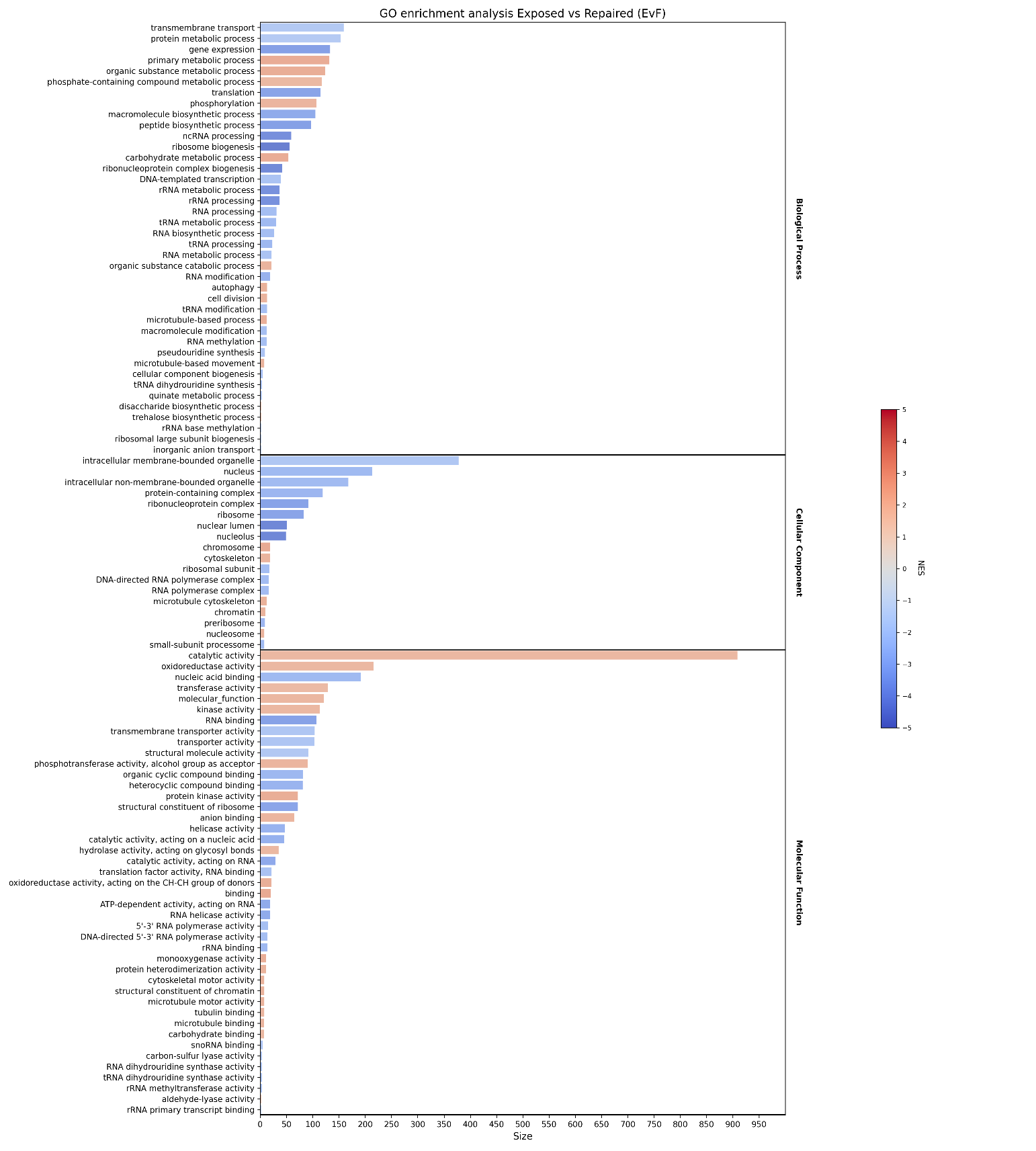


**Fig. S3** Shows the GSEA (Gene Set Enrichment Analysis) GO enrichment analysis performed with FungiFun3 for the Exposed vs Repaired sample. The figure summarizes the enrichment of each gene set and shows the significant ones padj<0.05. The genes are separated by ontology class: Biological process, Cellular component and Molecular function. The color of each bar represents the normalized enrichment score (NES) with for each pathway


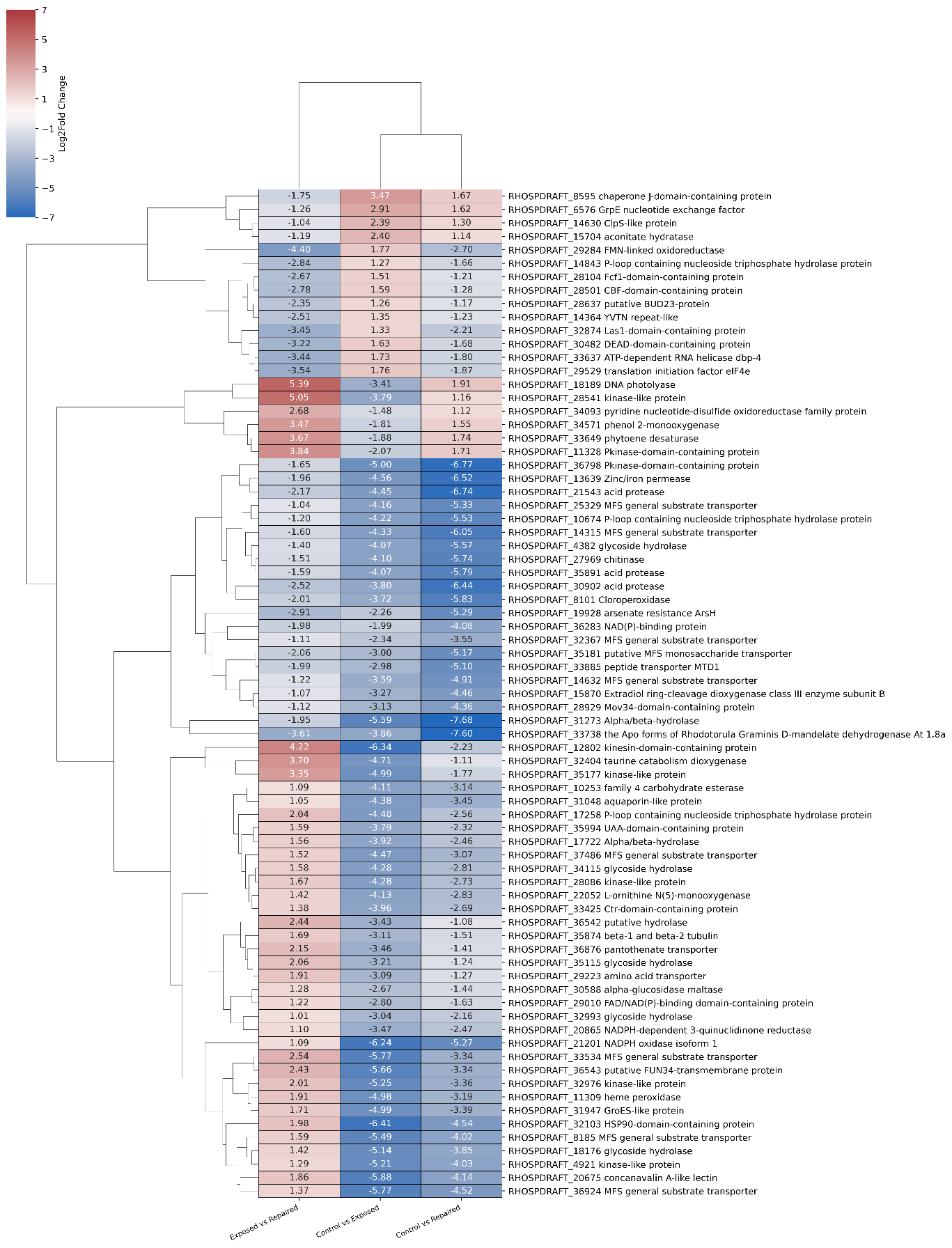


**Fig. S4** Clustered heatmap of the Control vs Exposed of GO enrichment of the differentially regulated genes in the analysis by FungiFun3


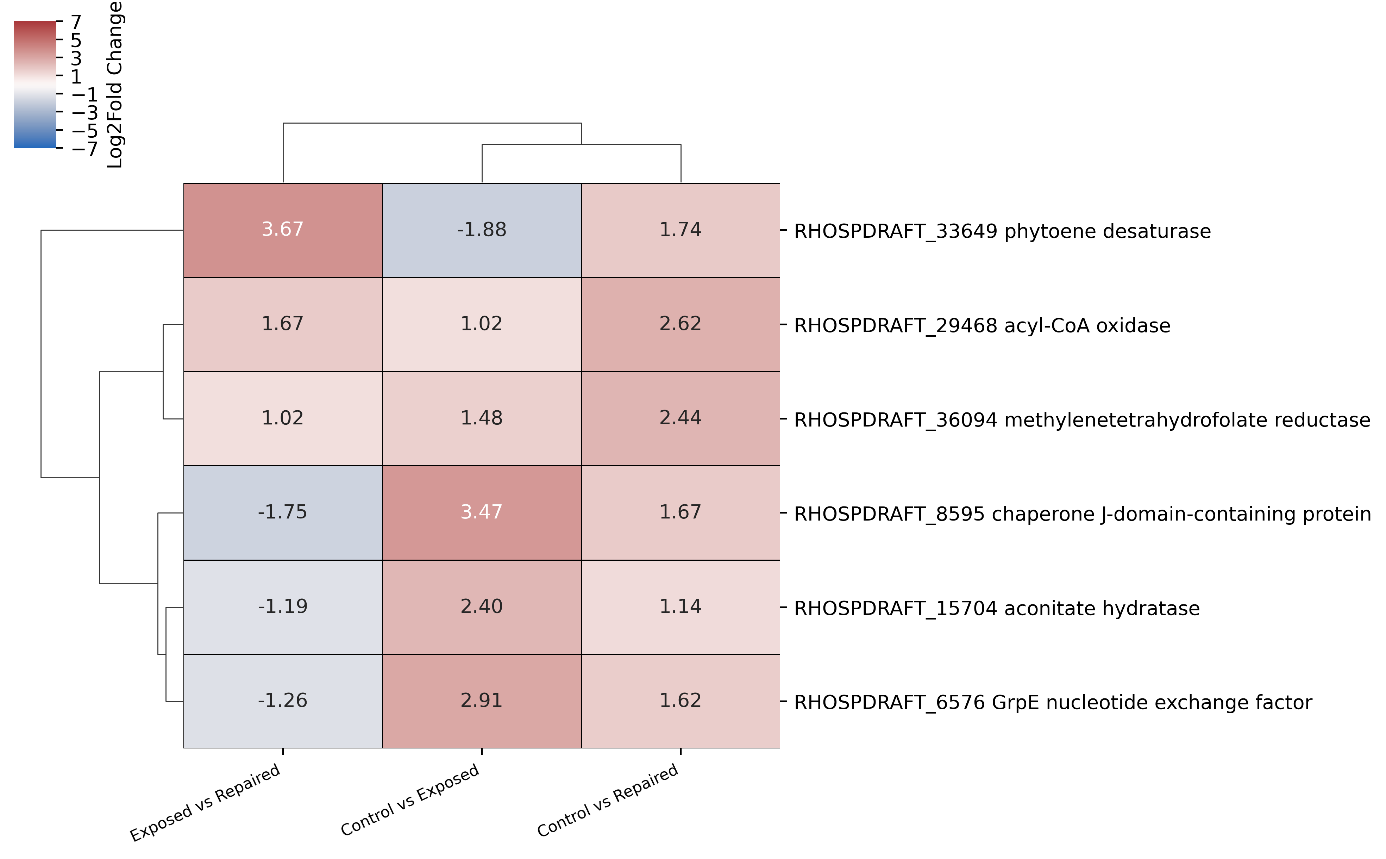


**Fig. S5** Clustered heatmap of the Control vs Repaired of GO enrichment of the differentially regulated genes in the analysis by FungiFun3


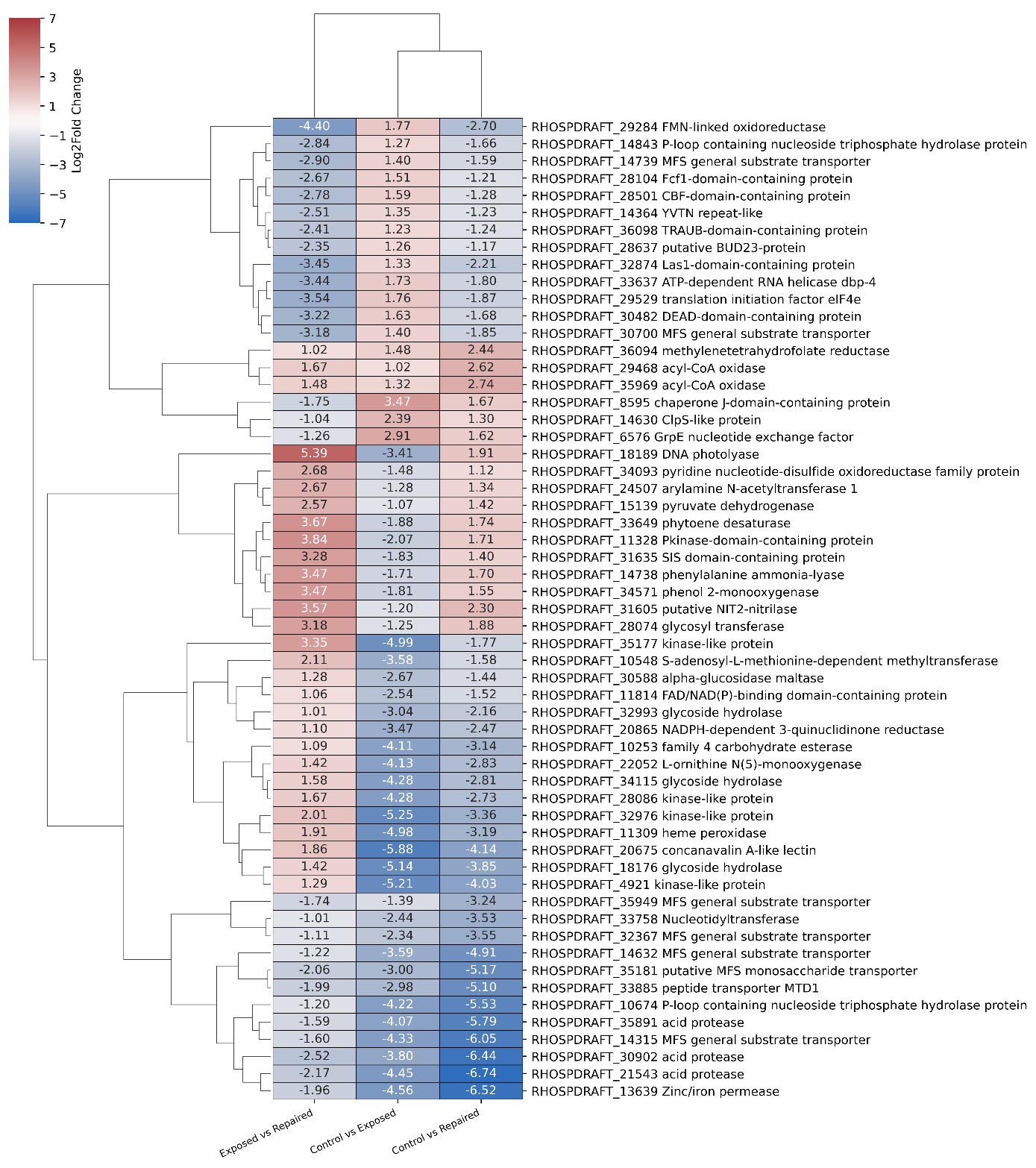


**Fig. S6** Clustered heatmap of the Exposed vs Repaired of GO enrichment of the differentially regulated genes in the analysis by FungiFun3
